# Pathological Chemistry

**Published:** 1858-03

**Authors:** 


ART. VI.—*Traité de Chimie Pathologique Appliqué à la Médecine Practique.* Par M. ALF. BECQUEREL et M. A. RODIER. Paris: Germer Baillière, 1854.

*Pathological Chemistry in its Application to the Practice of Medicine.* Translated from the French of MM. BECQUEREL and RODIER. By STANHOPE TEMPLEMAN SPEER, M.D., etc. etc. London: John Churchill, 1857.

FROM the deservedly high scientific character of its authors, the Pathological Chemistry of Becquerel and Rodier might, without examination, be presumed to merit an elevated position as a treatise, and it might be presupposed that by presenting a digest of our actual knowledge upon the subject it would fill a void which has hitherto existed in this department of medical literature.

The reader, however, who having formed such opinions, takes up this work with the confident expectation of having them confirmed, will, we think, be disappointed. Although it contains a great deal that is valuable, presented in a clear and concise manner, it is lamentably deficient both as a book of reference and as a guide to the analysis of pathological formations, or of the fluids and solids of the body in disease, and is, consequently, far from attaining to that standard of excellence which all scientific treatises of the present day must reach, if their authors desire to obtain for them a high appreciation from readers.

In order to show that we are not speaking loosely, it is only necessary to state that nowhere is any mention made of Trommer's test for diabetic sugar, of Liebig's for urea, or the slightest allusion made to the exact and convenient analytical processes by test-solutions, now so generally employed in the examination of the urine. The subject of urinary calculi is barely touched upon, and scarcely any directions given for their analysis.

From a neglect to take cognizance of recent discoveries, several inaccurate statements are made, which are calculated to lead to errors of serious importance. Thus, at page 278, (265 of the translation,) a table is given, which purports to show the mean quantity of urea excreted by a person in a state of health, during the twenty-four hours. This table is based upon observations of Becquerel, which were published in his *Séméiotique des Urines*, (p. 34,) over seventeen years since. The quantities stated are not one half those which are now obtained by improved processes, and yet no reference is made to any other results than those of Lecanu, which were published some time previously to Becquerel's.

At page 311, (295 of the translation,) the cells of the *torula cerevisiæ*, or, as MM. Becquerel and Rodier prefer to call them, "the globules of the sugar ferment," are stated some of them to be almost identical in appearance with the blood discs! No mention is made of the fungoid character of these bodies.

We might go on and point out many other inaccuracies and omissions, but we have no desire to multiply our citations. As we before observed, there is much in this work which is valuable. We may allude to the chapter on the blood, which is tolerably full and complete; but even here there are several instances of negligence, and but one method, that of MM. Becquerel and Rodier themselves, of analyzing this fluid is given.

In fact, the authors seem to have lost sight of most of the recent advances which have been made out of France in physiological and pathological chemistry. With that peculiar self-complacency in which Frenchmen are so wont to indulge, they appear to have imagined that those things not discovered in Gaul might have remained forever hid from mortal eye, and the world have been none the loser.

The translation of Dr. Speer is carefully, and even at times elegantly rendered. It is beautifully printed on fine white paper, in the style so usual with Churchill's publications that it has become commonplace to commend it. To those of our readers who are unacquainted with French, and who are desirous of possessing the *Chimie Pathologique*, we can confidently recommend this translation as being in every respect a faithful rendition of the original.

